# Alterations in functional connectivity of the anterior cingulate cortex associated with different levels of tau and amyloid-*β* deposition in patients with mild cognitive impairment

**DOI:** 10.3389/fnins.2026.1748031

**Published:** 2026-02-02

**Authors:** Wenzhang Qi, Yiming Ruan, Yue Tang, Darui Zheng, Qianqian Yuan, Chen Xue, Chaoyong Xiao

**Affiliations:** 1 Department of Radiology, The Affiliated Brain Hospital of Nanjing Medical University, Nanjing, Jiangsu, China; 2 Department of Radiology, The Affiliated Yixing Traditional Chinese Medicine Hospital of Yangzhou University, Wuxi, Jiangsu, China; 3 Department of Radiology, The Affiliated Chest Hospital of Nanjing Medical University, Nanjing, Jiangsu, China

**Keywords:** anterior cingulate cortex (ACC), cognize, fMRI, functional connectivity, MCI (mild cognitive impairment)

## Abstract

**Background:**

Three subgroups of mild cognitive impairment (MCI) may be identified based on the deposition of Aβ and tau proteins: A-T-, A + T+, and A + T-. The key hub for information processing and control, the anterior cingulate cortex (ACC), is essential for both healthy aging and MCI. The objective of this research is to systematically investigate changes in the functional connectivity (FC) of ACC subregions across different MCI subtypes.

**Methods:**

Overall, 54 A-T- patients, 28 A + T- patients, and 52 A + T + patients underwent FC analysis of ACC subregions. Correlation analyses were conducted to explore the relationship among pathological biomarkers, cognitive function, and FC changes in ACC subnetworks. The diagnostic utility of ACC-cortical FC in differentiating MCI subtypes was evaluated using receiver operating characteristic (ROC) curves.

**Results:**

Compared with the A-T- group, the A + T- group demonstrated reduced FC in the right precuneus and left dorsal ACC, whereas the A + T + group demonstrated increased FC in the left hippocampus, right precuneus and left dorsal ACC, the left superior frontal gyrus and right subgenual ACC. Compared with the A + T- group, the A + T + group demonstrated increased FC in the right precuneus and left dorsal ACC. The altered FC in ACC subnetworks was significantly correlated with pathological biomarkers in the cerebrospinal fluid. The ROC curve analysis suggested that changes in ACC FC effectively distinguished between the various pathological subtypes of MCI.

**Conclusion:**

In summary, different MCI subtypes have distinctive changes in the FC of ACC subregions, providing valuable insights into the mechanisms underlying MCI.

## Introduction

Mild Cognitive Impairment (MCI) is considered a transitional stage between healthy aging and Alzheimer’s disease (AD) (Jemimah et al., 2023). Numerous trajectories have been related to the clinical presentation of MCI (Winblad et al., 2004). The longitudinal progression from MCI to AD may be better predicted with the inclusion of biomarkers (Hinrichs et al., 2011). According to a new A/T/N binary biomarker classification model, amyloid-beta (A), tau (T), and neurodegeneration (N) are the three key biomarkers that comprise AD pathology (Jack et al., 2018). This model categorizes individuals into eight groups. Whereas abnormal A and T biomarkers (A + T + N − or A + T + N+) indicate AD, an abnormal A biomarker (A + T − N−) indicates Alzheimer’s pathology. The biomarkers A + T − N + indicate abnormalities that may suggest comorbidity or “AD with suspected non-Alzheimer’s pathology.” Individuals with A − are not considered to have AD continuum, and they may have biomarkers that indicate non-Alzheimer’s pathology (A − T + N−, A − T − N+, or A − T + N+) or normal biomarkers (A − T − N−) (Cousins et al., 2020). By facilitating the categorization of individuals based on their biomarker status, this classification approach expands existing knowledge of the factors contributing to cognitive impairment and provides fresh perspectives on the underlying processes of cognitive decline (Cheng et al., 2024).

Functional correlation (FC) analysis, a functional MRI technique, investigates deeper connections between brain regions by evaluating the correlation of fMRI signals across brain areas at rest (Xue et al., 2021). This approach has gained popularity in the study of cognitive processes, particularly in research on AD and MCI (Elberse et al., 2024). Both healthy aging and MCI are strongly related to the anterior cingulate cortex (ACC), which is critical to cognitive and emotional processing (Peng et al., 2021). The FC between the ACC and cortical regions is essential for preserving cognitive function; disruptions in this connectivity may contribute to cognitive impairments observed in neurodegenerative diseases (Huang et al., 2024). In addition, the structural changes in the ACC were closely related to tau protein deposition (Pezzoli et al., 2024). Therefore, based on the ATN classification, studying the functional changes in the ACC across different pathological subtypes of MCI may significantly aid in understanding the development of MCI. However, the differences in ACC-cortical connectivity amongst the several MCI subtypes identified by the AT(N) framework have not yet been thoroughly investigated.

Based on the AT(N) model, this study aims to explore the differences in the ACC-cortex FC across three groups of patients with MCI (A + T-, A + T+, and A-T- groups). Specifically, this study intends to investigate how different biomarker states (such as amyloid protein and tau) affect ACC function, as well as their relationship with cognition. By comparing these three groups, this research aims to identify alterations in the brain networks that might help diagnose MCI and predict disease progression by elucidating the neurobiological mechanisms underlying its subtypes.

## Methods

### Participants

Data for this study were sourced from the AD Neuroimaging Initiative (ADNI) database.^1^ The primary objective of ADNI is to validate AD biomarkers and investigate how these biomarkers, when combined with clinical and neuropsychological assessments, can monitor AD progression in individuals diagnosed with MCI or early-stage AD (Sakamoto et al., 2019). Comprehensive diagnostic criteria for MCI enrollment are available in the ADNI database.

For this study, data were collected from 226 patients with MCI whose cerebrospinal fluid (CSF) information was accessible through the ADNI2 and ADNI3 databases. After excluding patients with data missing and significant head movement, 134 patients remained. Following previously established criteria, a CSF Aβ42 concentration <977 pg./mL was considered abnormal, whereas that >24 pg./mL was used to define abnormal CSF phosphorylated tau (p-tau) levels (Ezzati et al., 2021; Vromen et al., 2023). Patients were divided into four groups based on these thresholds: (i) abnormal Aβ42 and p-tau (A + T+), (ii) abnormal Aβ42 with normal p-tau (A + T-), (iii) normal Aβ42 with abnormal p-tau (A-T+), and (iv) normal Aβ42 and p-tau (A-T-). The A-T + group was excluded because, based on the A/T/N framework, its classification did not correspond with the AD spectrum. Therefore, the final sample consisted of 54 patients in the A-T- group, 28 in the A + T- group, and 52 in the A + T + group.

### Ethics approval and consent to participate

Ethical approval for the ADNI study was obtained from the review boards of all participating institutions, and written informed consent was provided by the participants or their authorized representatives. For further information, please visit the ADNI website (www.adni-info.org).

### Cognitive function

Cognitive functioning was compared across groups using composite scores for situational memory (EM) and executive functioning (EF). EM and EF were further described in the Supplementary SI Methods.

### Pathological sample acquisition

Following the AD Association biomarker flowchart, CSF samples were collected. Aβ42, total tau (t-tau), and p-tau levels were measured using the INNO-BIAALZBio 3 immunoassay kit.

### MRI data acquisition

All MRI scans were acquired using a 3.0 T scanner, following unified scanning protocols obtained from various manufacturers, including Philips (Best, The Netherlands), General Electric (Cleveland, OH, USA), and Siemens (Munich, Germany). For more detailed information regarding scanning protocols, please refer to the following resources^2^: MRI Training Manual FINAL.pdf.^3^

### Functional data preprocessing

The preprocessing of fMRI data was conducted using the Data Processing and Analysis for Brain Imaging (DPABI) software^4^ within the MATLAB 2021b framework^5^ (Ruan et al., 2024). The detailed preprocessing is provided in the Supplementary SI Methods section.

### FC analysis

Three regions of interest were defined for each hemisphere in Montreal Neurological Institute (MNI) space: caudal ACC (A1; MNI = ± 5, −10, 37), dorsal ACC (A2; MNI = ± 5, 10, 33), and subgenual ACC (A3; MNI = ± 5.34, −4) (Kelly et al., 2009). Three-millimeter-radius spheres were used to symbolize each region. The FC between each region and the whole brain voxel was derived based on earlier research (Zhang et al., 2023). The specific method is explained in the Supplementary SI Methods.

### Statistical analysis

Statistical analyses were conducted using the Statistical Package for the Social Sciences (SPSS) software, version 25.0 (IBM, Armonk, NY, USA). Analysis of variance (ANOVA) and chi-square tests were conducted to assess differences in demographic characteristics, neurocognitive scores, and CSF pathological protein levels among the three groups (A-T-, A + T-, and A + T+). *Post-hoc* comparisons were conducted using Bonferroni correction, with a significance level of *p* < 0.05 for all tests.

After adjusting for age, sex, years of education and mean frame displacement (FD), a one-way ANOVA was conducted to evaluate FC differences in each insular ACC subregion (GRF corrected, voxel *p* < 0.005, cluster *p* < 0.05) (Zheng et al., 2025). *Post-hoc* comparisons were conducted using two-sample t-tests with the mask from the ANOVA analyses. Age, sex, years of education and mean FD were adjusted as covariates (*p* < 0.05). Additionally, the analysis was GRF adjusted, with a voxel *p* < 0.005 and cluster *p* < 0.05 (Zheng et al., 2025). Results report the clusters with voxel > 5.

A correlation analysis investigated the relationship between ACC functional status and clinical and physiological indicators. Specifically, the relationships between episodic memory (EM), executive function (EF), CSF pathological proteins (including Aβ, and pTau), and altered cortico-ACC FC were assessed using partial correlation analysis (with Bonferroni correction, *p* < 0.05).

The receiver operating characteristic (ROC) curve and the area under the ROC curve were used to evaluate the diagnostic value of altered FC of each ACC subregion in A + T- and A + T + groups. The results are reported as sensitivity and specificity.

## Results

### Demographic and neurocognitive characteristics

Table 1 represents the demographic and clinical data of all the study participants. The analyzed sample comprised 54 A-T- patients, 28 A + T-, and 52 A + T+. Compared to A-T- group, A + T- and A + T + group have significantly higher ages, but no significant age differences were found between A + T- group and A + T + group. There was no significant difference in age and education years among the three groups. No significant differences in MMSE and MoCA were observed between the three groups. Compared to A + T- group, A-T- and A + T + group have significantly lower EM scores, but no significant age differences were found between A-T- group and A + T + group. No significant differences in EF were observed between the three groups.

**Table 1:** Demographics and clinical measures of three groups.

General factors	A-T- (54)	A + T- (28)	A + T + (52)	*F*-value (*χ*^2^)	*p*-value
Age (years)	68.98 (7.70)	71.71 (7.27)	71.20 (7.14)	6.400	0.002
Gender (F/M)	20/32	13/15	20/29	0.375	0.688
PTEDUCAT	15.91	16.46	16.33	0.557	0.574
MMSE	28.06 (1.89)	28.18 (1.74)	27.39 (2.18)	1.495	0.228
MOCA	23.79 (3.06)	23.04 (2.52)	22.76 (3.61)	1.402	0.250
EM	0.49	0.30	0.46	4.615	0.012
EF	0.65	0.23	0.24	2.952	0.056
FD	0.13	0.11	0.13	0.762	0.469

Numbers are given as means (standard deviation, SD) unless stated otherwise. Scores reflect the number of correct items unless stated otherwise. Values for age derived from ANOVA; gender from chi-square test; all clinical measures from ANOVA with age and gender as covariates. MMSE, Mini-Mental State Examination; MOCA, Montreal Cognitive Assessment; EM, episodic memory; EF, executive function.

### FC analysis

The FD of all patients were less than 0.5, and there were no significant differences in mean FD among different groups.

**Figure 1:**
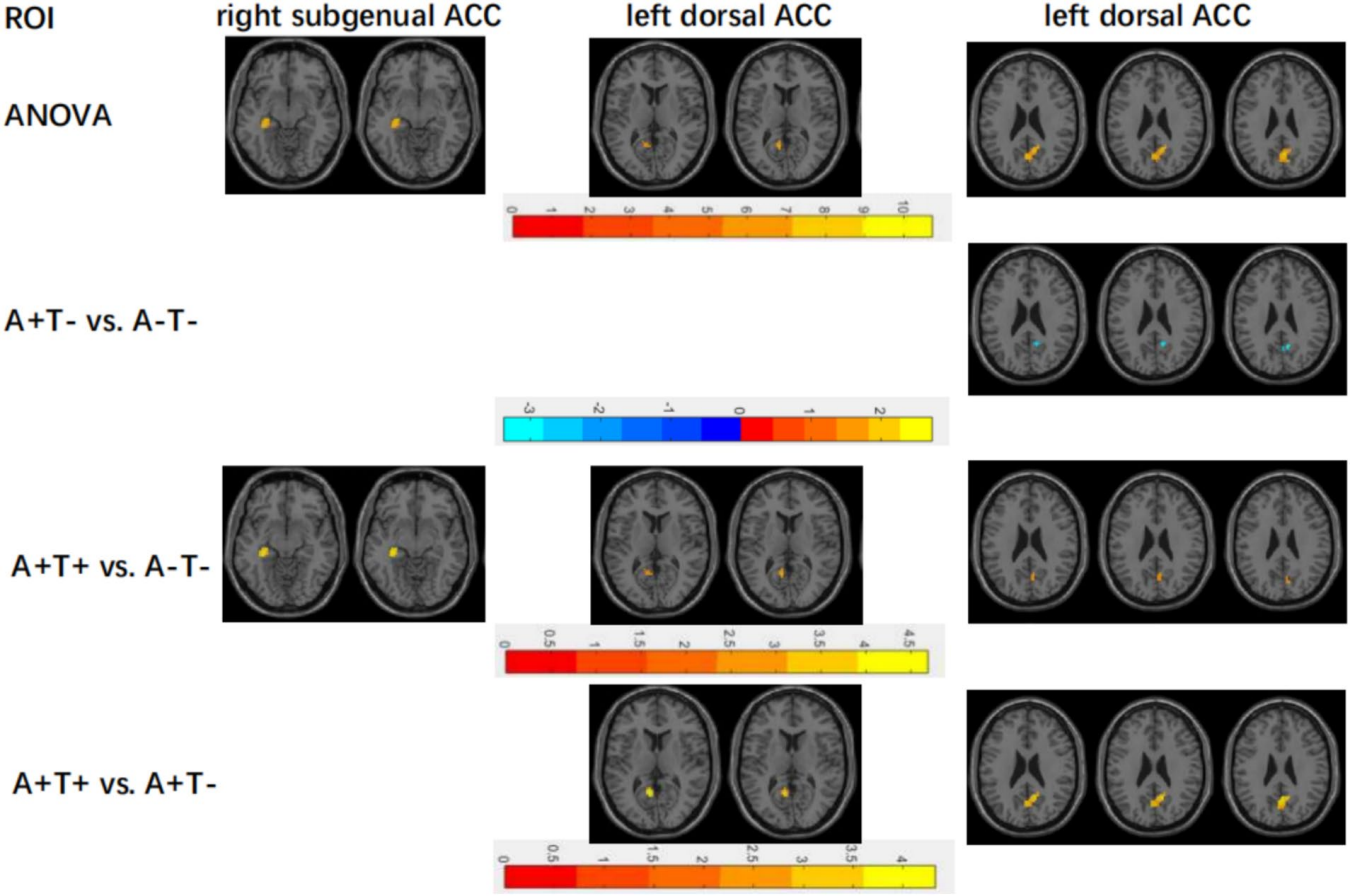
Differences in cortical FC between different ACC seed points across three subgroups. FC, functional connectivity; ACC, the anterior cingulate cortex; ROI, the region of interest; ANOVA, analysis of variance.

As shown in Table 2, in the left dorsal ACC network, ANOVA analysis showed that FC was significantly altered among three groups, including left hippocampus, left posterior cingulate and right precuneus. The A + T- group showed decreased FC in right precuneus compared to the A-T- group. (GRF corrected, voxel *p* < 0.005, cluster *p* < 0.05). The A + T + group showed increased FC in left hippocampus and right precuneus compared to the A-T- group and increased FC in right precuneus compared to the A + T- (GRF corrected, voxel *p* < 0.005, cluster *p* < 0.05). These results were obtained taking into account age, gender, years of education and the mean FD. (Figure 1).

**Table 2:** The difference of cortico-striatal functional connectivity among three groups.

Region(aal)	Peak MNI coordinate			F/t	Cluster number
	*X*	*Y*	*Z*		
Roi8 (left dorsal ACC)					
ANOVA					
L_ Hippocampus	−30	−24	−15	11.131	75
R_ Precuneus	6	−81	27	8.8846	77
A + T- vs. A-T-					
R_ Precuneus	12	−57	24	−3.3164	12
A + T + vs. A-T-					
L_ Hippocampus	−30	−24	−15	4.7634	75
R_ Precuneus	6	−81	27	3.8699	35
A + T + vs. A + T-					
R_ Precuneus	12	−57	24	3.7428	54
Roi9 (right subgenual ACC)					
ANOVA					
L_ Superior Frontal Gyrus	0	9	60	7.916	41
A + T- vs. A-T-					
A + T + vs. A-T-					
L_ Superior Frontal Gyrus	0	15	60	3.8875	41
A + T + vs. A + T-					

The *X*, *Y*, *Z* coordinates are the primary peak locations in the MNI space. Cluster size > 5 voxels in ANCOVA analysis, *p* < 0.05; Cluster size > 5 voxels in *post hoc* test, *p* < 0.05, GRF corrected; L, left; R, right.

In the right subgenual ACC network, ANOVA showed significant FC changes in left superior frontal gyrus (STG) among the three groups. Compared to the A-T- group The A + T + group showed increased FC in the left STG (GRF corrected, voxel *p* < 0.005, cluster *p* < 0.05). These results were obtained considering age, gender, years of education and the mean FD (Figure 1).

### Correlation analysis

**Figure 2:**
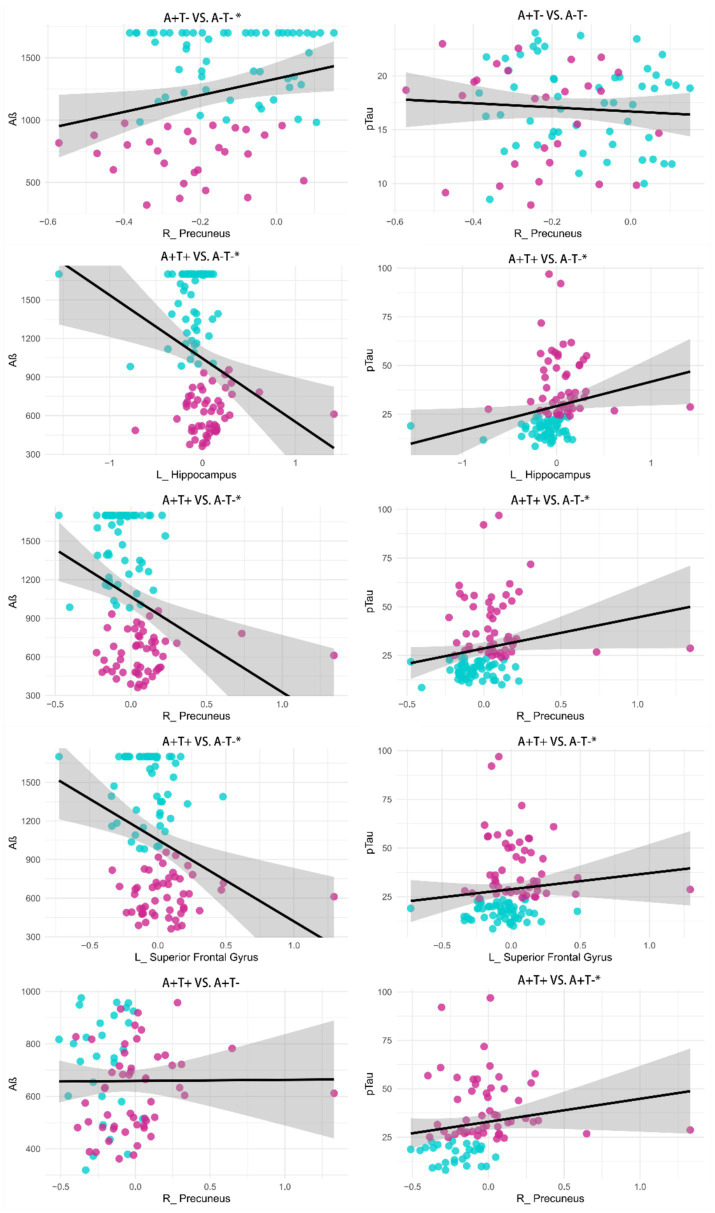
Results of associations between altered functional connectivity and pathological biomarkers. The Age, gender, years of education and the mean framewise displacement were used as covariates of results (Bonferroni corrected, *p* < 0.05). L, left; R, right, *, *p* < 0.05.

The correlation analyses demonstrated that compared to A-T-, the altered FC between the left dosal ACC and the right precuneus in A + T- was significantly associated with CSF Aβ (*r* = 0.328, *p* = 0.003). Compared to A-T-, the altered FC between the left dosal ACC and the left hippocampus in A + T + was significantly associated with CSF Aβ (*r* = −0.344, *p* < 0.001) and CSF tau (*r* = 0.289, *p* = 0.003), the altered FC between the left dosal ACC and the right precuneus in A + T + was significantly associated with CSF Aβ (*r* = −0.340, *p* < 0.001) and CSF tau (*r* = 0.254, *p* < 0.010), and the altered FC with the STG was significantly associated with both CSF Aβ (*r* = −0.340, *p* < 0.001) and CSF tau (*r* = 0.195, *p* = 0.050). Compared to A + T-, the altered FC between the left dosal ACC and the right precuneus in A + T + was significantly associated with CSF tau (*r* = 0.297, *p* = 0.009) (Figure 2). All results were adjusted using the Bonferroni correction, *p* < 0.05. There was no significant correlation found between altered ACC FC and EM and EF.

### ROC curve

**Figure 3:**
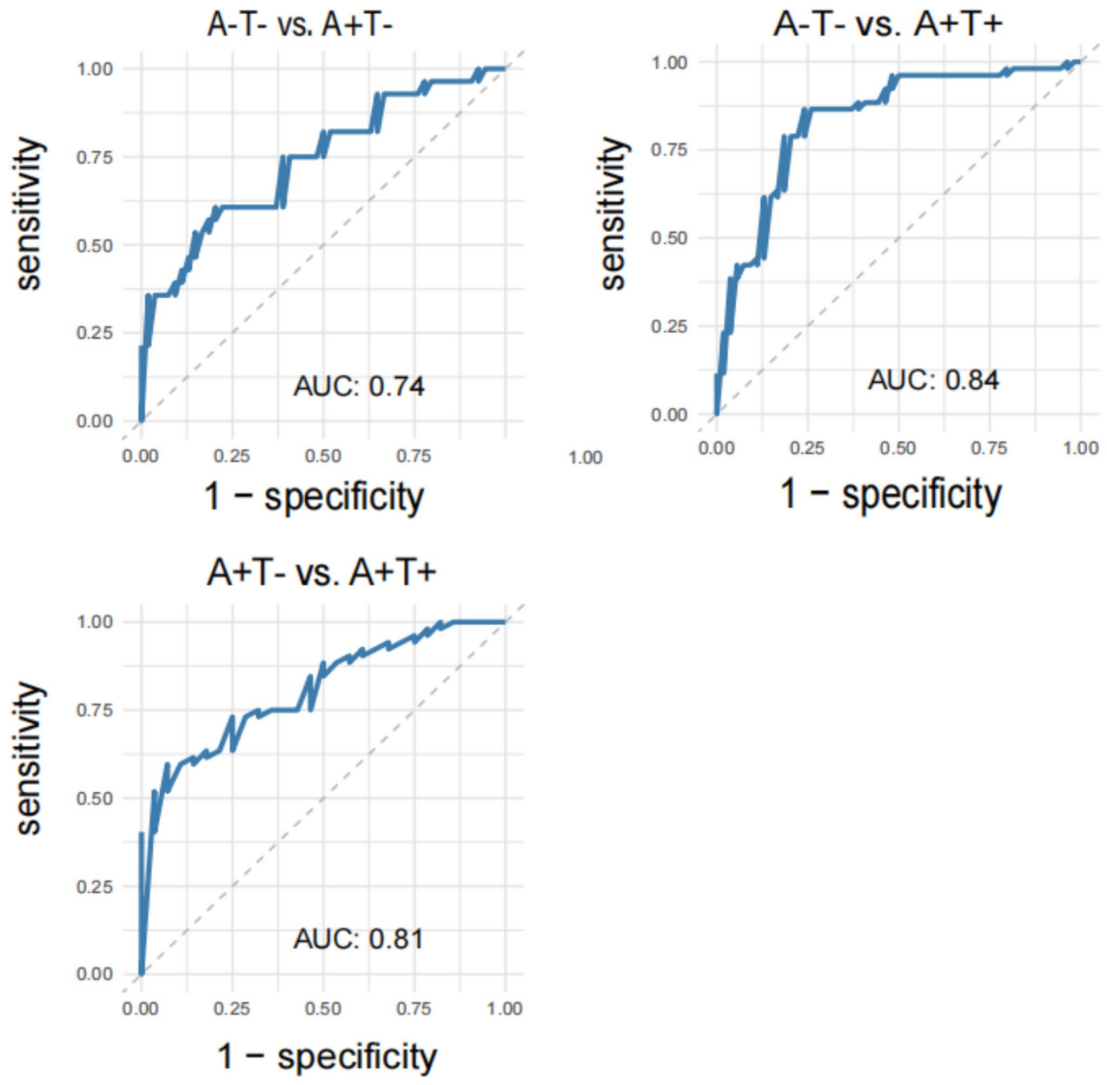
The ROC curves demonstrate significant differences in functional connectivity between different subtypes of MCI. ROC, receiver operating characteristic; MCI, mild cognitive impairment.

Figure 3 shows the evaluation of the diagnostic ability of cortical-striatal FC changes using the ROC curve. The AUC value for distinguishing the A-T- group from the A + T- group based on changes in ACC FC was 0.74. The AUC value for distinguishing the A-T- group from the A + T + group was 0.84, while the AUC value for distinguishing the A + T- group from the A + T + group was 0.81.

## Discussion

By comparing the differences among the A-T-, A + T-, and A + T + groups, this study explores the alterations in FC between the ACC subregions and the whole-brain network in MCI. MCI exhibited distinct ACC FC changes in several clinical subgroups, which were significantly correlated with CSF protein accumulation. This finding highlights the possible relevance of ACC subnetwork dysfunction in MCI progression by elucidating distinct patterns of FC alterations related to tau (T) and *β*-amyloid (A) pathology across time.

Specifically, both the A + T + and A + T-groups showed opposing patterns in the FC between the dorsal ACC and the precuneus, compared with the A-T- group. The precuneus, a central area of the DMN, is involved in several cognitive processes, such as self-awareness, spatial orientation, visual memory, and emotional processing (Cavanna and Trimble, 2006). Prior research has demonstrated a strong correlation between the progression of AD spectrum disorders and alterations in the precuneus (Koch et al., 2018). Both the early phases of AD and MCI have shown FC changes (Palmqvist et al., 2017). Furthermore, Aβ and tau accumulation may be strongly correlated with functional alterations in the precuneus (Buckner et al., 2009). The precuneus is more susceptible to Aβ accumulation than other cortical regions (Aghakhanyan et al., 2018). According to the correlation data, patients with AD and different pathological subtypes exhibited distinct ACC-precuneus FC changes, which may have been caused by the accumulation of pathogenic proteins. This result was consistent with previous research showing that the precuneus’s functional state is more susceptible to abnormal protein accumulation. The precuneus is closely related to the cingulate cortex, as a key region involved in cognitive and emotional processing (Lin et al., 2017). The ACC and precuneus exhibit comparable pathological changes in various diseases (Wee et al., 2016; Liu et al., 2014). Here, ACC-precuneus FC changes exhibited high sensitivity to Aβ and tau accumulation. The ACC-precuneus FC was proposed as a possible biomarker for protein alterations in the brain. The A + T + group’s increased ACC-precuneus FC may be a compensatory mechanism for brain function. AD improves connectivity of the important networks, such as the cingulate cortex and striatum, while decreasing internal connectivity within the default mode network (including the hippocampus and precuneus), compared with behavioral variant frontotemporal dementia (Zhou et al., 2010). Disrupted connectivity in the target network predicts enhanced intrinsic connectivity in the reciprocal network. Consequently, as a possible inter-network connection enhancement, ACC-precuneus connectivity may compensate for internal network connectivity dysfunction, indicating poor brain function. Notably, the A + T- group showed lower ACC-precuneus FC than the A-T-group, which was the reverse of the A + T + group. This may be related to the different effects of tau proteins on the brain. According to the “threshold model” proposed by Jack et al., tau pathology must act in tandem with Aβ accumulation to surpass the neural network compensation threshold, despite causing initial synaptic toxicity (Jack et al., 2018). There may be complex interactions between Aβ and tau, and some studies explore the Aβ/tau ratio as an indicator of pathological changes (Al-Ezzi et al., 2024). In this study, ACC-precuneus FC changes were strongly correlated with tau protein accumulation in the A + T + and A + T- groups (*r* = 0.199, *p* = 0.089). Therefore, tau protein accumulation may affect the brain’s self-regulation systems in addition to contributing to its toxic effects. This offers a fresh viewpoint on the physiological role of tau protein in AD progression and explains distinct clinical alterations in patients with various pathological subtypes of MCI and AD.

The A + T + group exhibited improved FC between the ACC and hippocampus, compared with the A-T- group; nonetheless, the A + T- group did not exhibit this difference. The hippocampus, a core region of the default mode network, is central to cognitive changes. Moreover, AD progression is strongly related to changes in its structure and function (Buckner et al., 2008). Therefore, changes in the hippocampus attract more attention. Improved hippocampal preservation may be attributed to the A + T + group’s stronger ACC-hippocampus connectivity. MCI presents various patterns of brain atrophy. Depending on the type and degree of brain atrophy, MCI can be categorized as classical AD type, hippocampal-preserved type, limbic atrophy type, or small atrophy type (Ekman et al., 2018). Statistical analysis suggested that the A + T + group had a higher prevalence of hippocampal-preserved and small atrophy types, suggesting that A + T + MCI may have greater preservation of hippocampal structures. Notably, this pattern is entirely reversed by the AD stage. In a recent study, patients with AD in the A + T + group exhibited significant bilateral hippocampal atrophy, which was significantly correlated with a higher risk of cognitive decline than patients in the A + T-group (Pan et al., 2025). Therefore, the A + T + group’s stronger ACC-hippocampus FC may be related to this structural preservation and suggest a greater likelihood of early intervention. ACC FC is a viable detection node because the ROC curve demonstrates its effectiveness at differentiating between A-T-, A + T-, and A + T + groups. Moreover, patients with AD commonly exhibit impaired FC between the PCC and hippocampus (Berron et al., 2020). In reciprocal functional areas, the increased ACC-hippocampus connectivity may serve as a compensatory mechanism. Interestingly, a strong correlation was observed between changes in ACC-hippocampus connectivity and Aβ and tau protein levels, suggesting that these proteins may regulate connectivity. In addition to the hippocampus, the A + T + group showed FC changes between the dorsal ACC and the STG. The STG is a key brain region involved in higher-order processes, such as emotional recognition and cognitive control (Dixon et al., 2017). These patients may show worse cognitive and emotional performance because of variations in ACC-STG connectivity.

Finally, this study demonstrated that the distribution of the brain cortex related to the ACC varied between the two hemispheres, indicating some cross- and asymmetry in the FC changes in the ACC-cortex. Except for the ACC-hippocampus connection, all alterations primarily affected the ACC side that was connected to the contralateral cortex. This finding may be related to the ACC’s neuroanatomy and function. Anatomically, the hippocampal-cingulate pathway, which is also a component of the limbic system, passes ipsilaterally through the cingulum bundle, whereas the corpus callosum fibers of the anterior cingulate provide contralateral connections (Hopper and Vogel, 1976). The ipsilateral ACC-hippocampus connectivity may be more sensitive to functional changes in the ACC owing to the strong anatomical relationship (Wang et al., 2025). Furthermore, bilateral brain regions usually need to cooperate to conduct higher cognitive activities (Wu et al., 2024). Cognitive decline is strongly related to abnormal inter-hemispheric connectivity, which is a hallmark of brain dysfunction. Functional changes in the ipsilateral ACC-hippocampus are one possible compensatory mechanism for this impaired brain function (Wu et al., 2020). Therefore, utilizing the ACC as a node to analyze the distinct changes between the two hemispheres may identify related pathway for identifying patients with impaired brain function.

However, this study has some limitations. First, because the study was cross-sectional, it could not clarify the causal relationship or dynamic changes between FC and Aβ/tau over time. Second, the study only used functional imaging data, lacking structural and diffusion images, which limits the interpretation of certain functional changes. Furthermore, individual fMRI connectivity measurements have limited test–retest reliability in cross-sectional designs. Third, this experiment faced issues with the relatively modest and unbalanced data, which may affect statistical power. Additionally, as it only used a single database from ADNI, future studies should include more centers and balanced subgroup samples to improve result reproducibility. Finally, this study primarily used correlation-based linear methods to describe the relationship between pathological biomarkers and functional connectivity. However, there may also be non-linear interactions, including indirect connections across multiple brain regions. The further research was needed.

In conclusion, different pathological subtypes of MCI within the ATN framework exhibited distinct FC in the cingulate cortex network, which were significantly associated with Aβ and tau accumulation in the CSF. These results suggesting associations between protein pathology and brain FC in patients with MCI and identifying related markers for the early diagnosis of different MCI subtypes.

## Data Availability

The datasets presented in this study can be found in online repositories. The names of the repository/repositories and accession number(s) can be found at: http://adni.loni.usc.edu.

## Footnotes

1
http://adni.loni.usc.edu

2
http://adni.loni.usc.edu/wpcontent/uploads/2010/05/ADNI2

3
http://adni.loni.usc.edu/wp-content/uploads/2017/07/ADNI3-MRI-protocols.pdf

4
http://rfmri.org/DPABI

5
http://www.mathworks.com/products/matlab/

## Group members of the Alzheimer’s Disease Neuroimaging Initiative

Data used in preparation of this article were obtained from the Alzheimer’s Disease Neuroimaging Initiative (ADNI) database (adni.loni.usc.edu). As such, the investigators within the ADNI contributed to the design and implementation of ADNI and/or provided data but did not participate in analysis or writing of this report. A complete listing of ADNI investigators can be found at: http://adni.loni.usc.edu/wp-content/uploads/how_to_apply/ADNI_Acknowledgement_List.pdf.
