# Supplementary material for: Alterations in functional connectivity of the anterior cingulate cortex associated with different levels of tau and amyloid-β deposition in patients with mild cognitive impairment
Source: Front Neurosci. 2026 Feb 2;20:1748031. doi: 10.3389/fnins.2026.1748031 (PMC12908466; doi:10.3389/fnins.2026.1748031)
Supplement: Supplementary file 1 [file Data_Sheet_1.docx]

Supporting Information

Supplementary Method

1.Cognitive function

The cognitive functioning of all subjects was assessed using a standardized neuropsychological test battery, including the Mini Mental State Examination (MMSE), Montreal Cognitive Assessment (MOCA). The episodic memory function (EM) was evaluated by the composite score of MMSE, the Rey Auditory Verbal Learning Test, the Alzheimer Disease Assessment Scale-Cognitive and Logical Memory. The executive control function (EF) was assessed by the composite score of clock drawing, WAIS-R Digit Symbol, Category Fluency, Trails A & B and Digit Span Backwards. All neurocognitive assessments were available on the ADNI website(<https://ida.loni.usc.edu/pages/ac,cess/studyData)>.)

2. Functional data preprocessing

The preprocessing of fMRI data was conducted using the Data Processing and Analysis for Brain Imaging (DPABI) software (http://rfmri.org/DPABI) within the MATLAB 2021b framework (<http://www.mathworks.com/products/matlab/>).

The process began by discarding the first 10 volumes to enhance the stability of the MRI signal. Subsequently, slice timing correction and head movement correction were applied. Subjects' images were excluded if the translation or rotation exceeded 3 mm or 3°, respectively. At the same time, calculated the frame displacement (FD) and the mean FD each subject. FD threshold was set to 0.5, and the frames with displacement greater than the threshold were excluded. The images were then spatially normalized to the MNI echo-planar imaging template and resampled to a default voxel size of 3 x 3 x 3 mm³. To minimize confounding effects on the dependent variable, nuisance covariates—such as 24 motion parameters, white matter signal, and cerebrospinal fluid signal—were regressed out. Remove the global signal to enhance signal quality. High-frequency noise was reduced by applying a 6 x 6 x 6 mm full-width at half maximum (FWHM) Gaussian smoothing filter. Finally, a temporal bandpass filter (0.01–0.08 Hz) was applied to eliminate low-frequency drifts and high-frequency noise.

3.FC analysis

These regions were represented by spheres with a 3 mm radius centered on the specified coordinates. The average time course within each seed region was extracted and correlated with all voxels across the entire brain to generate the FC of the ACC. The Pearson correlation coefficients (r) between ROI activation and each voxel were then Fisher-Z transformed. Finally, for each subject, four z-score maps were generated to represent the intrinsic FC patterns.
